# A compartmental model of an identified *Drosophila* larval motoneuron for investigating functional effects of ion channel parameters

**DOI:** 10.1186/1471-2202-13-S1-P66

**Published:** 2012-07-16

**Authors:** Cengiz Günay, Logesh Dharmar, Fred Sieling, Richard A Baines, Astrid A Prinz

**Affiliations:** 1 Dept. Biology, Emory University, Atlanta, Georgia 30322, USA; 2 Biomedical Engineering Dept., Georgia Inst. Tech. and Emory Univ., Atlanta, Georgia, USA; 3 Life Sciences, University of Manchester, Manchester M13 9PT, UK

## 

*Drosophila* is a powerful genetic model system for investigating neuronal function. Several important membrane ion channel genes, such as voltage-gated sodium and potassium channels, were first identified and isolated in the fruit fly. Technical advances in experimental methods have recently made possible direct electrophysiological recording of ionic currents in central neurons, allowing the genetic advantages of this system to be applied to analysis of cellular and circuit function and homeostasis.

An important open question is the functional effect of channel splice variants, which have recently been found in *Drosophila* neurons [1]. The composition of splice variants, which result in the observed sodium current, changes in an activity-dependent manner in seizure mutant *Drosophila* (personal communication with W-H Lin, R Marley, and RA Baines) and may be the underlying cause of the increase of the persistent component of the sodium current [2]*.* Because of experimental limitations, computational modeling is essential for understanding the functional implications of this change.

We previously presented a computational approach to determine the full set of biophysical parameters of the sodium channel splice variants [3]. We then replicated combinations of these splice variants observed in flies and inserted them into a minimal, isopotential spiking model neuron with transient and persistent sodium, delayed-rectifier, and A-type potassium channels. This isopotential model was limited to reproducing only some firing properties of real neurons because of the morphological distribution of ion channels in these neurons. Specifically, sodium channels that are responsible for action potential initiation are located far from the soma where the recordings are made.

In the present work, we improve this model neuron by including morphological details. We take the morphological information from identified larval aCC abdominal dorsomedial motoneurons, which innervate the dorsal muscles [4]. A two-compartment version of the model is used to assess effects of changing sodium channel properties. This neuron model allows investigating the effect of sodium channel splice variants by varying half-activation and inactivation voltages and ratio of a persistent component to mimic changes observed in sodium channel current properties in seizure mutants. We further analyze the effect that changes in synaptic input observed in seizure mutants have on the output neuronal activity.
